# Structural regulation and dynamic behaviour of organelles during plant meiosis

**DOI:** 10.3389/fcell.2022.925789

**Published:** 2022-10-25

**Authors:** Aybars Koç, Nico De Storme

**Affiliations:** Plant Genetics and Crop Improvement Lab, Department of Biosystems, KU Leuven, Leuven, Belgium

**Keywords:** meiosis, organelles, mitochondria, plastids, cytoskeleton

## Abstract

Eukaryotes use various mechanisms to maintain cell division stability during sporogenesis, and in particular during meiosis to achieve production of haploid spores. In addition to establishing even chromosome segregation in meiosis I and II, it is crucial for meiotic cells to guarantee balanced partitioning of organelles to the daughter cells, to properly inherit cellular functions. In plants, cytological studies in model systems have yielded insights into the meiotic behaviour of different organelles, i.e., clearly revealing a distinct organization at different stages throughout meiosis indicating for an active regulatory mechanism determining their subcellular dynamics. However, how, and why plant meiocytes organize synchronicity of these elements and whether this is conserved across all plant genera is still not fully elucidated. It is generally accepted that the highly programmed intracellular behaviour of organelles during meiosis serves to guarantee balanced cytoplasmic inheritance. However, recent studies also indicate that it contributes to the regulation of key meiotic processes, like the organization of cell polarity and spindle orientation, thus exhibiting different functionalities than those characterized in mitotic cell division. In this review paper, we will outline the current knowledge on organelle dynamics in plant meiosis and discuss the putative strategies that the plant cell uses to mediate this programmed spatio-temporal organization in order to safeguard balanced separation of organelles. Particular attention is thereby given to putative molecular mechanisms that underlie this dynamic organelle organization taken into account existing variations in the meiotic cell division program across different plant types. Furthermore, we will elaborate on the structural role of organelles in plant meiosis and discuss on organelle-based cellular mechanisms that contribute to the organization and molecular coordination of key meiotic processes, including spindle positioning, chromosome segregation and cell division. Overall, this review summarizes all relevant insights on the dynamic behaviour and inheritance of organelles during plant meiosis, and discusses on their functional role in the structural and molecular regulation of meiotic cell division.

## Introduction

Meiosis is a highly specialized cell division that involves a single DNA replication, followed by two subsequent rounds of chromosome segregation. It results in the production of four daughter cells, each of them having half of the chromosome number of the mother cell. In plants these newly formed haploid daughter cells undergo two or more rounds of mitotic cell division to form gametes that have specialized roles in reproduction, i.e. with fusion of gametes during pollination and fertilization leading to the next generation of plants. Meiosis is thus a crucial step in the life cycle of sexually proliferating plants. In general, angiosperms exhibit two types of meiotic cell division programs, namely the successive and the simultaneous-type of cell division, and these principally differ in the specific timing of cytokinesis. In successive cytokinesis the separation of the cytoplasm occurs consecutively after each meiotic division. This results in an intermediate dyad stage after meiosis I (MI) that consists of 2 cells residing within the pollen mother cell wall, divided by a callose wall. This type of meiotic cell division occurs both in male and female sporogenesis of most monocotyledonous angiosperms and some gymnosperms, as well as in female sporogenesis in dicotyledonous plants. In simultaneous cytokinesis, cell wall formation only takes place after both nuclear divisions have occurred, i.e. thus after telophase II, with the formation of distinct cell plates between the four haploid nuclei formed (Albert, Raquin et al., 2011; Tchorzewska 2017). This type of meiotic cell division program occurs exclusively in male sporogenesis of Bryophyta, Pteridophyta and dicotyledonous angiosperms. The type of meiotic cell division determines the morphology of produced tetrads. Meiocytes with a successive-type of cytokinesis produce tetragonal, linear, T-shaped, Z-shaped or decussate tetrads, whereas meiocytes that undergo simultaneous-type of cytokinesis may result in tetrahedral, rhomboidal, tetragonal or decussate tetrad configurations (Albert, Raquin et al., 2011).

Research on plant meiosis has primarily been conducted on the dynamics of chromosomal structures and cytoskeletal figures (spindles, RMA, *etc.*). However significantly less is currently known about the concurring patterns of organelle movement in the cytoplasm. Evidence from non-plant systems, mostly mammalian and yeast cells, suggests that a highly organized organelle partitioning mechanism is operational in meiosis, i.e. similar to the mechanism of nuclear genome separation, to maintain the correct and balanced inheritance of organelles. Behaviour of cytoplasmic organelles during meiotic cell division has been studied in several eukaryotic organisms and this has revealed that, for proper cell division and further functioning of resulting spores, it is crucial that the organelles are evenly segregated to the resulting daughter cells, with several regulatory mechanisms mediating this balanced segregation (Mishra and Chan 2014). Initially it was assumed that the organelles follow simple patterns of positioning throughout plant meiosis, showing a random scattering in the cytoplasm that is passively affected by cytoplasmic streams (Mahlberg 1964). However, as several studies in model systems have revealed distinct cytoplasmic grouping and aggregations of certain organelles during several stages of meiosis, it has now become clear that plants, like all other eukaryotic organisms, host unique patterns of organelle behaviour throughout meiotic cell division indicating for active regulatory mechanisms that mediate spatio-temporal behaviour of organelles (Rodkiewicz 1988).

A typical plant cell is composed of the nucleus, mitochondria, plastids, and the endomembrane system, which includes the endoplasmic reticulum (ER), the Golgi apparatus, the vacuole, peroxisomes, the plasma membrane and endosomes. Organelles are specialized subcellular structures that perform specific functions in the cell, and in most cases are bound by a lipid membrane. Mitochondria, plastids, the endoplasmic reticulum, the Golgi apparatus, vacuoles and endosomes are among the membrane-bound organelles, whereas ribosomes are an example of non-membrane-bound organelle group. Plastids and mitochondria contain their own genome and for this reason, they can only be replicated from existing organelles. Mitochondria, perhaps the most studied organelle type, are known for their function in energy production, i.e., by generating ATP molecules from glucose *via* the oxidative phosphorylation pathway. However mitochondria also have crucial roles in other metabolic and cellular reactions, such as clustering of iron-sulphur groups (Stehling, Wilbrecht et al., 2014), and apoptosis (Tait and Green 2010). Another important type of organelles in the plant cell are plastids. These are a group of plant-specific organelles that can be divided into several major groups based on morphology, colour, and function. The chloroplast, one of the most well-known plastids, is a photosynthetic organelle that is named after its green colour. In addition to the inner and outer membranes, chloroplasts have a third internal membrane structure, named the thylakoid membrane. This membrane is crucial, as it harbours the electron transport system in plants and plays a role in the generation of ATP. Both light-dependent and light-independent stages of photosynthesis take place in the chloroplasts, where light is utilized to start a chain of reactions that eventually results in the production of sugar and other organic molecules that are necessary for the cell’s metabolism. In flowering plants, and particularly in aging leaves and fruits, chloroplasts can differentiate and convert into chromoplasts, which come in a variety of different colours and hence contribute to pigmentation, although their exact function in the cell metabolism is not clear. Additionally, in leaves that do no longer benefit from photo-assimilation, such as upon aging or shading, chloroplasts can lose their photosynthetic capacity and get re-purposed into so-called gerontoplasts. Lastly leucoplasts, non-pigmented plastids, are present in the non-photosynthetic organs of the plant, such as roots, and can be further sub-categorized into amyloplasts, proteinoplasts and elaioplasts. In general, these different types of plastids are used as a storage structures for organic compounds.

Mitochondria and plastids have retained many features from their presumed bacterial ancestors, according to the endosymbiotic theory, such as the capability to produce ATP *via* specialized elements on their inner membrane, a proteome that is highly similar to that of proteobacteria, and most importantly, a double membrane structure. Additionally, mitochondria can go through fusion and fission events, which involves the physical merging of several mitochondria to form one large mitochondrion, and the separation of a mitochondrion into several mitochondria, respectively (Chan 2012). Mitochondria generally maintain a balance between these events to keep a steady number of individual mitochondrial units, although in some cell types, such as in onion leaf mesophyll cells, extensive mitochondrial fusion occurs leading the formation of a single enlarged interconnected membraned structure. Both mitochondria and plastids possess their own genome, referred to as the mitochondrial (mtDNA) and plastid DNA (ptDNA), respectively, which encodes for elements that are significant for bio-energetic functioning. These circular DNA molecules are spatially organized and partially condensed to yield specific mtDNA- or ptDNA-protein complexes, called nucleoids (Pyke 2010; Mishra and Chan 2014). When all the copies of mtDNA or ptDNA within a cell are identical, the cytoplasmic state for that specific organelle is termed homoplasmic. Heteroplasmy, on the other hand, is defined as the presence of two or more different variants of mitochondrial or plastid genomes in a cell. Considering the maternal inheritance of the cytoplasm in most sexually reproducing organisms, plant heteroplasmy was initially associated with pathological mutations or was mostly found in genetically modified plant populations. However, currently it is viewed as a common situation in plants, mostly resulting from small-scale mutations or paternal leakage of mtDNA/ptDNA (Woloszynska 2010).

Next to mitochondria and plastids, plant cells also contain an extensive endomembrane system which is composed of the endoplasmic reticulum (ER), the Golgi apparatus, and the vacuole as most essential components. In most plant cells, the ER is organized as a web-like structure with membranous layers, that stretches from the nucleus to the cell cortex. It mainly functions as a network that mediates the movement of proteins and other molecules. The Golgi apparatus facilitates the processing and targeted delivery of several important molecules to specific regions in the cell. It plays a distinctive role in cell division in plants because of its necessary function in cell plate formation (Wick 1991; Staehelin and Hepler 1996). Initially, the Golgi body was thought to be the sole actor providing building components for the formation of cell plate, but evidence was brought to light suggesting that material from the cell membrane that has been endocytosed also contributes to the biosynthesis of the plate structure (Staehelin and Hepler 1996). The Golgi apparatus is composed of building blocks, called Golgi stacks, that undergo fusion and tethering to hence give rise to various sized organelles. The vacuole, in terms of volume, is the largest organelle in plant cells and can occur in a wide variety of shapes and forms. Its main function in plants is to organize the removal of waste products, and as an extension, to maintain the water balance within the cell (Greer, Diamond et al., 1982; Garcia-Herdugo, Gonzalez-Reyes et al., 1988).

In non-dividing plant cells cytoplasmic organelles are not fixed and move around, following the actin/myosin-dependent cytoplasmic streaming with distinct localization patterns (Tominaga and Ito 2015). Their patterned movements, outside of cell division, were found to be caused by the nucleus being moved around rapidly in response to external stresses, such as pathogenic and environmental effects. In this regard, targeted relocation of chloroplasts, due to changes in light intensity, were seen to cause similar patterned movement of organelles (Mathur 2020). It was also reported that under specific stresses, small organelles like mitochondria, peroxisomes and lipid droplets surrounding larger organelles form clustered organelle structures (Frederick and Newcomb 1969; Jaipargas, Mathur et al., 2016). It was also noted that the increase of turgor pressure in plant cells causes formation of narrow cytoplasmic sleeves that limit the movement of larger organelle types, like chloroplasts, while smaller organelles, as well as vesicles and other components in the cytoplasm, continue to move freely (Mathur 2020).

In mitotically dividing plant cells, most of the organelles in the cytoplasm of the daughter cells are inherited from the mother cell, as they cannot be *de novo* synthesized within the daughter cell. Upon division, the inherited organelles grow and propagate to fulfil the required cellular amounts, before they start functioning within the cell (Westermann 2014). Balanced segregation of organelles during plant mitosis, like in other organisms, is hence critical for proper cellular proliferation and functioning, and is therefore strictly controlled in a spatio-temporal way *via* several molecular mechanisms (Nagata 2010; Kianian and Kianian 2014; Tchorzewska 2017). Despite this extensive knowledge on organelle behaviour in plant mitosis, current insights on organelle behaviour in plant meiosis are rather limited, raising the question whether organelle segregation is also relevant in this reductional-type of cell division and whether similar mechanisms operate to mediate organelle dynamics throughout meiosis I and II. In this review, we compile the current understanding of the dynamic behaviour of different types of organelles in plant meiosis in the context of balanced organelle inheritance, and elaborate on the molecular mechanisms that regulate this programmed motility together with the functional role of specific organelle structures in regulating key processes in meiotic cell division.

## Temporal dynamics and localisation of different types of organelles during meiosis

### Mitochondria and plastids

In most eukaryotic cells, mitochondria undergo fusion-fission events that are paralleled to and coordinated with the cell cycle. These events are essential for growing and dividing cells to provide them with sufficient numbers of mitochondria, i.e. mainly to foresee in the required energy supply (Westermann 2014; Strzyz 2021). In addition, these fusion-fission events also are thought to be important for the repair of damaged mitochondria and for intermixing DNA and proteins between the mitochondria present in the cell (Sheridan and Martin 2010; Strzyz 2021). Mitochondrial fusion mainly occurs during transition from G1 stage to S stage and involves the coalescing of two or more mitochondria by a physical merging of their outer membranes. This facilitates the transport of compounds between these organelles (Sheridan and Martin 2010). Mitochondrial fission, on the other hand, mainly occurs during the G2 and M phases of the cell cycle and is achieved by the cleavage of interconnecting membranes between mitochondria. In *S. cerevisiae*, mitochondrial fission is mediated by the cytosolic protein DRP1, a member of the dynamin protein family that generally functions as helical scaffold and uses energy release from GTP hydrolysis to remodel and constrict membrane intermediates (Udagawa, Ishihara et al., 2014). Current evidence suggests that DRP1 binds to the mitochondrial membrane at specific constriction sites and promotes fission by aiding in the construction of microtubules that lead fission events (Sheridan and Martin 2010). Suppression of *DRP1* usually leads to the arrest of cell cycle at G2/M phase, resulting in enlarged mitochondrial networks and occasional aneuploidy, indicating a functional role for mitochondrial fission in cell cycle regulation (Kianian and Kianian 2014). Throughout mitotic cell division in plants, mitochondria come at different morphologies and structural configurations, i.e. with a single mitochondrion or multiple mitochondria fused together in the G1 stage, after which they often merge and elongate during the transition from G1 to S stage, most likely to support the high metabolic demand during DNA replication. Next, at the following G2 and M phases, mitochondria go through rapid fission events and form several individual units that are randomly dispersed throughout the cytoplasm. This cell-cycle dependent mitochondrial organization and proliferation is essential for organelle inheritance during cell division, as it forms a passive mechanism to promote equal distribution of mitochondria into the daughter cells. Finally, after cell division, some of the mitochondria regain their elongated form when entering the G1 stage (Mishra and Chan 2014; Chacko, Mehta et al., 2019). In contrast, during meiotic cell division in plants, like in several other eukaryotic species, mitochondria show a more orchestrated spatio-temporal patterning dynamics, indicating for active mechanisms regulating their spatial localization throughout the reductional cell division. During prophase I and metaphase I stages mitochondria group together at the two poles of the cell and are attached to the cell cortex *via* anchoring proteins (Rodkiewicz 1988; Wakai, Harada et al., 2014). Later, in telophase I, mitochondria become disconnected from the cell periphery after which, in many species, they relocate towards the equatorial region of the dividing cell where they group with several other types of organelles, i.e. including plastids, Golgi and endomembrane vesicles, in a band-like structure spanning the whole length of the cellular midzone. This ‘organelle band’ is a typical feature of meiocytes with a simultaneous-type of cytokinesis where it is maintained at the equatorial plane until late anaphase II as a sort of physical barrier that separates the two haploid chromosome sets throughout MII. Next, at the end of meiosis II, the mitochondria, like other organelles, are released from the organelle band and are re-positioned between the four newly formed haploid nuclei to create an X-shaped structure that delineates the four cytoplasmic domains and that forms a structural framework to mediate subsequent cytokinesis (Bednara, Stobiecka et al., 1977; Rodkiewicz 1988). In meiocytes with a successive-type of cytokinesis, this equatorial organelle band is generally not formed in MI, though with some exceptions, indicating that it is not a critical structure for mediating cell plate formation after the first meiotic division.

Plastids are morphologically flexible and go through a form of elongation and shrinking during mitotic and meiotic cell division. While not as dynamic as mitochondrial events, it was reported that plastids generally take an elongated form during the early prophase stage of division, and keep this differentiated form until the end of prophase I, before they revert back to their original morphology (Dickinson 1981; Busby 1988). In lower plants, such as bryophytes and cryptogams, meiosis initiates from a sporocyte mother cell that only contains one single plastid and this type of meiosis is therefore referred to as a monoplastidic type. In these cells, microtubule systems physically associate with plastids, mediate their division, and subsequently position the four resulting daughter plastids in a tetrahedral configuration to hence assign meiotic cell polarity and mediate spindle orientation and development. More detailed information about the dynamic behaviour of plastids during monoplastidic type meiosis comes from several moss species and other lower land plants, like *Funaria hygrometrica* and *Timmiella barbuloides*. In early stage meiosis, plastids elongate, starting in early prophase I and continuing on through mid-prophase I, until they completely envelope the nuclear membrane. The plastids then rotate and take a perpendicular configuration at specific regions along the nuclear envelope, while remaining anchored to the nucleus, thereby repositioning towards the edges of a tetrahedron that assign meiotic cell polarity. After mid-prophase I, the plastids start to shrink and eventually co-localize with mitochondria at the poles of the cytoplasm (Busby 1988). In higher plant species, the meiotic progenitor cells contain many plastids and the associated cell division is therefore categorized as polyplastidic type (Brown and Lemmon 1990). Plastids hereby show a highly similar dynamics as the mitochondria, as described above. Indeed, in meiocytes of angiosperms and gymnosperms plastids are initially found in a grouped state in prophase I and at the beginning of metaphase I, specifically localizing at the two opposite poles of the cell. Later, towards the end of telophase I, plastids are repositioned towards the cell equatorial region, and co-localize with other organelles to form a band-like structure. In meiocytes with a successive-type of cytokinesis, this band is only detectable in some plant species and it is not maintained as it is replaced by a cell wall at the end of meiosis I. Contrary, in meiocytes with a simultaneous-type of cytokinesis, this organelle band is always formed and remains intact until the end of telophase II to physically assign and delineate the two cytoplasmic regions in meiosis II (Medina, Risueno et al., 1981; Rodkiewicz 1988).

In plants, like in other eukaryotes, meiosis and mitosis show different spatio-temporal patterning of plastids and mitochondria during cell division, indicating for distinct regulatory mechanisms mediating organelle dynamics in both division types. During somatic cell division of *S. cerevisiae* and *N. tabacum* epidermal cells, mitochondria and plastids stay grouped at the opposite sides of the cell until the end of cytokinesis, and this is mediated through protein anchoring mechanisms. Only once the division is completed, separated organelles are dispersed in the cytoplasm, hence indicating for a seemingly passive segregation process (Hanson and Sattarzadeh 2008; Jongsma, Berlin et al., 2015; Chacko, Mehta et al., 2019). On the other hand, in plant meiotic cell division, i.e. following a similar polar organelle grouping in prophase I and metaphase I, mitochondria and plastids join together with Golgi and other organelles at the equatorial region of the dividing cell at the end of telophase I to form a clustered group or even a plate-like structure, also called the organelle band (Gorsich and Shaw 2004). This highly organized organelle patterning in meiosis indicates for the involvement of more active regulatory processes as compared to somatic cell division, and putative underlying mechanisms are further discussed below.

### Endoplasmic reticulum

The ER is a large organelle structure that is mainly composed out of a network of lacy membranes and that is typically subdivided in two subunits: the rough endoplasmic reticulum (RER) and the smooth endoplasmic reticulum (SER). In non-dividing stages of the cell, the ER is physically connected to the nucleus with its membranes continuously flowing over into the outer nuclear membrane. Studies in pea (*Pisum sativum* L.) revealed that in meiotic prophase I most of the ER in the cytoplasm changes from rough to smooth and additionally shows a concomitant increase in development of Golgi cisternae (Medina, Risueno et al., 1981). It is hypothesized that these morphological changes are related to the formation of large sized vacuoles in the subsequent cell division stages, resulting from connected functions of the SER and the Golgi apparatus (Medina, Risueno et al., 1981). However, further evidence for this mechanistic link are still lacking. In the following stages of meiosis, i.e., up till cytokinesis, smooth ER is found closely associated with plastids, and often surrounding them. This may be tied to the specific elongation behaviour of plastids seen in lower plants during meiosis (Brown and Lemmon 1990), thus indicating a putative role of the ER in regulating plastid configurations. In support of this, recent studies on organelle morphologies have shown that the ER acts as a mediator of the differentiation of certain cellular organelles, including plastids, mitochondria, and peroxisomes. It has been demonstrated for example that the ER interacts with mitochondria for several minutes and exchanges matrix proteins, before the linked mitochondria enter into a fusion event (Mathur 2020). Perhaps, a similar dynamics occurs in plastids as well, where the ER potentially facilitates plastid elongation through supplementation of constitutional proteins. At the end of meiotic division, the ER re-localizes together with plastids, and is mostly found within the (mini-)phragmoplasts, a complex assembly of microtubules and filaments that acts as a framework for cell plate formation in plant cells, between segregated sets of chromosomes. In line with this, during mitotic cytokinesis of *Nicotiana tabacum* L cells, ER was observed to associate with the newly formed cell plate in the division region (Gupton, Collings et al., 2006). This behaviour of the ER was initially hypothesized to aid in the formation of the cell plate, i.e., by providing a pocket-like structure in which Golgi-derived vesicles containing cell wall building blocks can accumulate through fusion (Staehelin and Hepler 1996). However, in later studies, 3D tomography analyses revealed that the ER is not present in the cellular midzone during the early vesicle fusion stages but only joins the cell plate later (Segui-Simarro and Staehelin 2006). It is therefore currently hypothesized that the close proximity of the ER to the cell plate aids in the transport of lipid membranes towards the cellular midzone to support cell plate maturation and cell wall formation (Segui-Simarro and Staehelin 2006)*.* Unfortunately, there are currently no reports available documenting the behaviour of the ER during plant meiosis. Due to the lack of evidence on meiosis that proves otherwise, the ER is currently thought to follow a stochastic dynamics during MI and MII that involves fragmentation followed by scattered dispersion throughout the cytoplasm in both telophase I and telophase II, similar as seen in mitotic cytokinesis of *Allium cepa* (Warren and Wickner 1996).

### Golgi apparatus

The number and volume of Golgi structures in a plant cell largely differs depending on cell type and species. Additionally, the volume of Golgi bodies was seen to increase throughout the life cycle of several plant cells. In *Arabidopsis*, for example, the total Golgi volume in the cell doubles in the G2 phase of the mitotic cell cycle (Segui-Simarro and Staehelin 2006). A similar increase was also observed in *Allium cepa* cells between prophase and telophase stages of mitotic cell division (Garcia-Herdugo, Gonzalez-Reyes et al., 1988). During interphase, Golgi bodies are stacked and are scattered in the cytoplasm. In prophase of mitosis, Golgi stacks are fragmented and in metaphase and anaphase, small Golgi fragments disperse through the cytosol to guarantee balanced inheritance. After cytokinesis, Golgi structures are reassembled in each daughter cell around the MTOCs (Table 1) (Jongsma, Berlin et al., 2015). Strikingly, in plant meiosis, the Golgi apparatus exhibits a different morphological dynamics and subcellular positioning. In the pachytene stage of meiosis in pea (*Pisum sativum* L.), Golgi seem to decrease both in number and in the level of activity (Medina, Risueno et al., 1981). It was suggested that at this stage, Golgi bodies undergo disassembly into Golgi stacks before reaching metaphase I, and then again reassembly later at telophase I (Oke and Suarez-Quian 1992). Interestingly, two large accumulations of Golgi stacks were observed in *Pisum* meiosis during telophase I, namely at the opposite poles of the spindle (Nebenfuhr, Frohlick et al., 2000). These accumulations were hypothesized to function as possible reserves for Golgi-derived vesicles that could aid in the correct reassembly of the partitioned Golgi bodies (Oke and Suarez-Quian 1992). It is also possible that this behaviour ensures that at least some copies of each Golgi unit (also called cisternae) are present in each of the resulting daughter cells after cytokinesis, as each cisterna has its own complex protein composition and thus cannot be simply replaced by another one (Hermo, Green et al., 1991). Studies in tobacco (*Nicotiana tabacum* L.) revealed that meiotic cells later in development accumulate a large amount of Golgi bodies in early meiosis II with the Golgi stacks specifically localizing on the equatorial midzone during pro-metaphase II, forming an organelle group referred to as the Golgi belt (Medina, Risueno et al., 1981; Nebenführ 2007). Interestingly, this belt has not been observed in *Arabidopsis*, though this could be due to overall lower amount of Golgi stacks. The Golgi belt is specific for simultaneous-type of meiotic cell division, and is presumed to mark the site of MI division in the cell with its localization likely being tied to Golgi’s function in packaging and transporting components that are essential for cell plate formation. However, this currently remains unproven as experimental studies in tobacco BY-2 cells have shown that the disruption of Golgi stacks with inhibitor agents does not inhibit or interfere with mitotic cell plate formation (Nebenfuhr, Frohlick et al., 2000). Depending on the plant species, the Golgi belt, together with mitochondria, plastids and other small organelles group together at this stage to form a transient plate structure, i.e. the organelle band, that separates the two cytoplasmic domains resulting from MI before actual cytokinesis at the end of MII (Rodkiewicz 1988; Sheahan 2007). In higher plants, thousands of Golgi stacks were observed during the G2 stage of somatic cell division, whereas in yeast this number only amounts to 20 (Griffing 1991). This excessively high number implies that random dispersion of Golgi stacks should suffice for balanced partitioning during cell division in plants, hence indicating that cellular inheritance of Golgi bodies most likely occurs *via* a passive mechanism (Griffing 1991; Warren and Wickner 1996). However, it should be noticed here that there is currently not enough evidence to suggest that random or organized partitioning of Golgi bodies are mutually exclusive occurrences, so it is plausible that also active mechanisms underlie cellular inheritance of Golgi. Moreover, with plant meiosis showing a different spatio-temporal patterning of Golgi as compared to mitotic division, it is yet unclear whether and to what extent the inheritance of Golgi during the two subsequent meiotic divisions occurs *via* the same mechanism as in somatic division.

**TABLE 1:** A list of abbreviations and their definitions used in the text.

Abbreviations
MECA, mitochondria-ER cortex anchor
MTOC, microtubule organizing center
NE, nuclear envelope
PM, plasma membrane
QMS, quadripolar microtubule system

### Vacuole

In *Arabidopsis* meiosis, the vacuole enlarges between prophase I and metaphase I, and this occurs at such a high rate that the majority of the meiotic cell’s volume becomes vacuolar at the onset of chromosome segregation in late metaphase I (Medina, Risueno et al., 1981). At this stage, the vacuole is mostly localized towards the peripheral region of the cell. The concomitant absence of the vacuole from the centre of the cell defines the phragmosome; i.e. the cellular region in which cytokinesis occurs in plant cells (Nebenführ 2007). In addition to this structural positioning, the early vacuole expansion in meiosis is hypothesized to have two cellular functions: 1) to aid in the reduction and then in the restoration cycle of ribosomes, that occurs between prophase I and the end of meiosis I as found in lily (*Lilium henryi* L.), maize (*Zea mays L.*), and pea (*Pisum sativum* L.)*,* and 2) to guarantee an even distribution of the cytoplasm between resulting daughter cells (Medina, Risueno et al., 1981). Interestingly, immediately preceding this vacuolization stage, the ER was observed to alter from rough to smooth, and the number of small vesicles in the cell was seen to increase significantly (Medina, Risueno et al., 1981), suggesting that the vacuolization process during early meiosis is regulated by smooth ER and Golgi activity.

After metaphase I, the vacuole breaks down into smaller parts which are then randomly scattered in the cytoplasm. However, this breakdown was not observed in all plant species, as for example in tobacco (*Nicotiana tabacum L.*), most likely due to a higher level of vacuolization (Griffing 1991; Segui-Simarro and Staehelin 2006). Until its fragmentation in metaphase I or later, the vacuole forms tubular extensions that reach into the phragmosome, thereby forming physical contact points that bind the two parts of the vacuole across the cell. This large tubular vacuolar network is hypothesized to play a crucial role in the transport of essential proteins and molecules during meiotic cell division (Medina, Risueno et al., 1981; Nebenführ 2007; Brownfield, Yi et al., 2015).

### Peroxisomes and ribosomes

Organelles that are not part of the endomembrane system have not received as much attention as the rest, particularly regarding their behaviour during meiotic cell division. In this group, most research has been performed on peroxisomes found in male meiotic cells of onion (*Allium cepa* L.) and leek (*Allium porrum* L.) which exhibit the simultaneous-type of cytokinesis. It was hereby seen that peroxisomes are randomly distributed in the cytoplasm throughout the first meiotic division, but then accumulate in the central area of the cell between interkinesis and metaphase II. This accumulation was preceded by the formation of the phragmoplast and an actin filament organization in the same region, suggesting a putative role of these structures in the targeted localization of peroxisomes (Collings, Harper et al., 2003). As a similar movement was observed for organelles that contribute to the formation of the equatorial organelle band, this could mean that peroxisomes also form a part of this band, or at least are closely associated with it. Studies in onion (*Allium cepa*) showed that during mitotic cytokinesis, when the phragmoplast structure expands outwards, clusters of peroxisomes follow behind while being closely associated with the newly formed cell plate (Collings, Harper et al., 2003; Nebenführ 2007). This behaviour was hypothesized to help with the production of hydrogen peroxide radicals at the cell plate, which are known to function in cell wall hardening, or perhaps, also have a role in the lipid recycling in the membrane (Nebenführ 2007). However, this specific localization of peroxisomes at the newly formed cell plate was not observed in neither meiotic or mitotic cells of Arabidopsis (*Arabidopsis thaliana L.*) or tobacco (*Nicotiana tabacum* L.), implying that peroxisome accumulation during cell plate formation may vary between species or cell types, and may be highly species-specific (Collings, Harper et al., 2003).

Ribosomes are complex molecules that are composed of different ribosomal RNA subunits, and that function in the cell as regulators of protein synthesis. Their biogenesis takes place both in the nucleus and in the cytoplasm of eukaryotic cells, and they are mostly found freely dispersed in the cytoplasm, while some of them are attached to the RER (Dickinson and Heslop-Harrison 1977). In meiosis of Henry’s lily (*Lilium henryi* L.), maize (*Zea mays* L.)*,* and pea (*Pisum sativum* L.), the population ribosomes in the cytoplasm was found to start decreasing as early as from the leptotene stage and from then on showed variation in subcellular location. This gradual decrease continues towards the end of prophase I, at which ribosomes are no longer observed (Medina, Risueno et al., 1981). Only after completion of meiosis I, ribosomes are again generated and re-accumulate within the cytoplasm in a highly stochastic fashion. Interestingly, the exact timing of this depletion-accumulation cycle differs slightly across plant species, with the re-accumulation in *Pisum* for example occurring later as compared to *Lilium* (Nebenführ 2007). So far, there is no evidence suggesting co-localization of ribosomes with the organelle band, and this indicates that ribosomes are likely passively distributed or are positioned in meiosis II *via* other mechanisms. As ribosomes can be generated *de novo* in the newly formed daughter cells through nucleus-encoded ribosomal RNAs, it is unlikely that resulting spores would suffer from having insufficient ribosomes at the end of meiosis, for example due to an unbalanced segregation in MI or MII.

### Organelle clustering and equatorial plate formation during meiosis

Mitochondria, plastids, and other organelles typically group with each other at specific stages during meiotic cell division. Studies in multiple eukaryotic organisms, including brown rat (*Rattus norvegicus*) and budding yeast (*Saccharomyces cerevisiae*), have shown that during meiotic prophase I and metaphase I, organelles cluster at opposite sides within the cell with a specific positioning near the chromosomal plate at late metaphase I (Rodkiewicz 1988; Westermann 2014; Brownfield, Yi et al., 2015; Sawyer, Joshi et al., 2019), and that this positioning is established and maintained through physical linkage with the cell cortex *via* anchor proteins (Wakai, Harada et al., 2014; Chacko, Mehta et al., 2019). Based on studies in mammalian oocytes it has been proposed that this subcellular positioning aids in locally providing the necessary ATP for the high-energy demanding processes of spindle formation and chromosome segregation (Li and Fan 1997). In plants, this polar organelle grouping in early meiosis I has also been observed in bryophytes, pteridophytes, gymnosperms and angiosperms that exhibit a simultaneous-type of cytokinesis, putatively serving a similar function in MI chromosome segregation. However, there are not many reports of similar polar organelle grouping in plants that exhibit a successive-type of cytokinesis. A notable example of this is reported in the monocot widow’s tears (*Tinantia erecta*), in which plastids and mitochondria were observed to group at the cell poles during early prophase I (Marciniec, Zieba et al., 2019).

**FIGURE 1:**
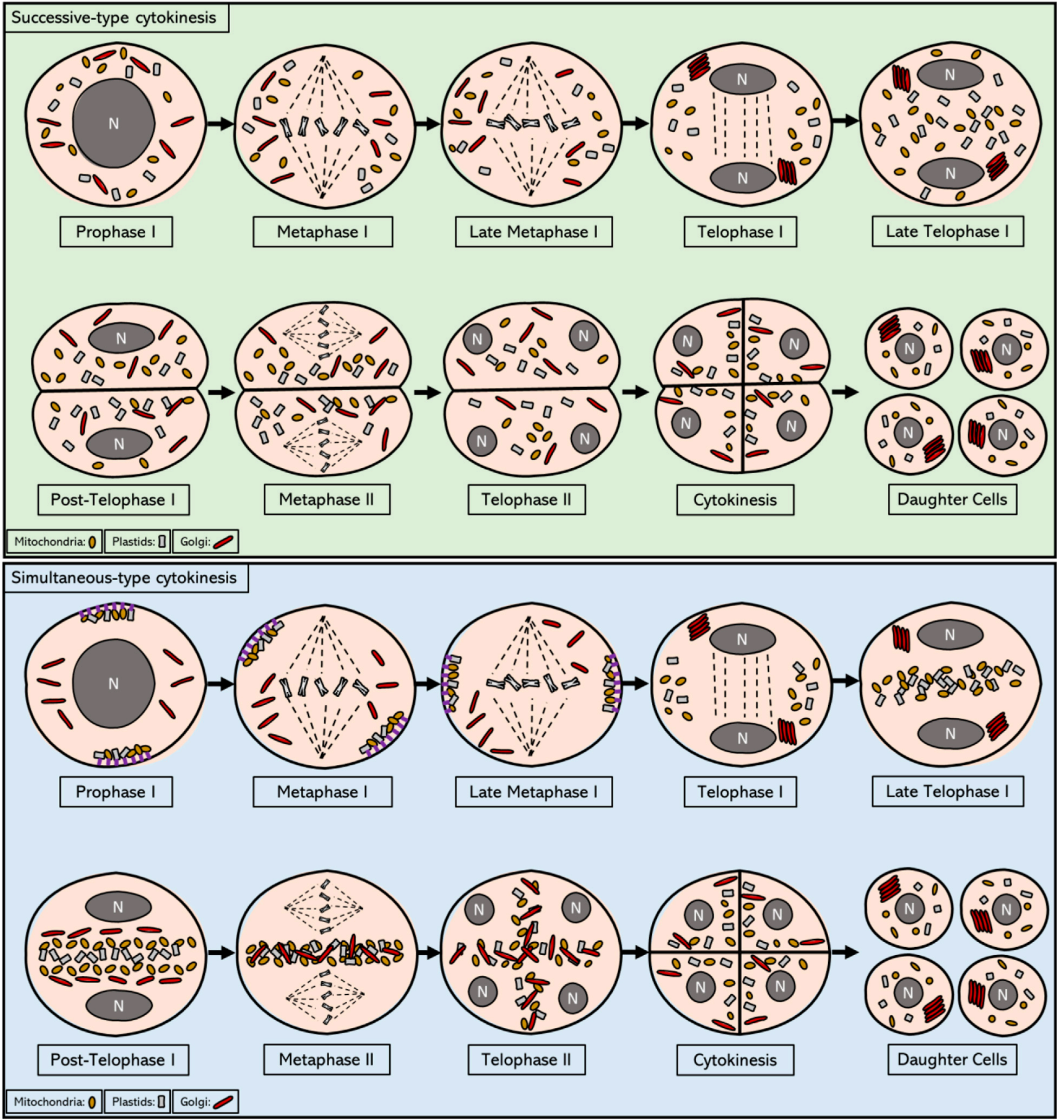
Intracellular localization of mitochondria, plastids, and Golgi from prophase I to telophase II in meiotic cells that exhibit successive and simultaneous type of cytokinesis. In the most prevalent form of successive cytokinesis, mitochondria, plastids and Golgi bodies are dispersed in the cytoplasm from prophase I until the end of metaphase I. In telophase I, Golgi stacks reassemble and move towards the newly formed nuclei. By the end of telophase I, first callose wall is formed and the pollen mother cell enters the dyad stage, consisting of 2 cells within the same cell wall. In some plant organisms with successive-type cytokinesis, as for example in *Larix europea,* a short formation of an equatorial organelle band is observed during this stage that disperses as soon as callose wall is formed at the end of telophase I. The organelles remain scattered in the cytoplasm until the end of telophase II, when the second callose wall is formed, yielding the four haploid daughter cells. Overall, the organelle movements during this process are reminiscent of mitotic cell division, in which an active regulation of organelle motility seems to be non-existent. In plant meiocytes with a simultaneous-type of cytokinesis, mitochondria and plastids are grouped on the opposite sides of the cell towards the end of prophase I and are attached to cell cortex *via* anchor proteins, while stacks of Golgi bodies are found scattered in the cytoplasm in a disassembled form. During metaphase I all organelle types start migrating towards the central midzone, and by the end of metaphase I they are grouped in the cytosol near the chromosomes. During telophase I, Golgi stacks reassemble and localize in close proximity to the newly formed nuclei, whereas mitochondria and plastids start occupying the cytoplasmic space at the equator of the cell. At the end of telophase I, Golgi stacks also join the equatorial group thereby forming a thick organelle band. After telophase I and before meiosis II, each type of organelle is shortly found to be grouped with each other (separated from different organelles) and ordered in a linear fashion across the cell equator. This linear ordering is transient and is quickly disturbed prior to metaphase II, most likely due to the cytoplasmic streaming resulting from the biogenesis and activity of the spindles. The distinct organelle band is maintained at the central midzone throughout metaphase II and anaphase II. After anaphase II, all type of organelles are released from the central organelle band and become repositioned to the internuclear cytoplasmic regions, hence again forming distinct organelle layers that are stationed between each of the four haploid nuclei. At the end of meiosis II, these internuclear organelle clusters are physically segregated into the resulting daughter cells through the establishment of the cell wall during cytokinesis.

Following MI chromosome segregation in meiocytes with a simultaneous-type of cytokinesis as well as in some with a successive-type of cytokinesis, organelles move towards the equatorial midzone to form by the end of telophase I a plate-like structure that expands across the whole central region of the cell (Rodkiewicz 1988). This equatorial plate is referred to as the ‘organelle band’ and consists of mitochondria, plastids, the Golgi apparatus, endomembrane vesicles, and possibly several other small organelles (Sheahan 2007; Brownfield, Yi et al., 2015) (Figure 1). The composition of organelles that cluster at the cellular midzone during telophase I was found to differ slightly between plant species, with the most unusual organization observed in moss, in which plastids do not join the central organelle band at all (Rodkiewicz 1988). In most plant species, with some notable exceptions like plants with a successive-type of meiotic cytokinesis and also some plant species that exhibit a simultaneous-type of meiotic cell division, like rush skeletonweed (*Chondrilla juncea* L.), the grouped organelles remain clustered in this equatorial midzone throughout interkinesis and the complete second meiotic cell division (Koscinska-Pajak 2003). It is currently not clear how this transient organelle band structure manages to remain in a stable position at the cell centre, despite a continuous pressure from cytoplasmic streams and chromosome movements. Only a slight bend in the shape of the organelle band was observed during this period in the European terrestrial orchid (*Epipactis helleborine*) (Bednara, Stobiecka et al., 1977). This central organelle grouping lasts until the end of telophase II, after which it loses its integrity during transient cell plate formation and is replaced by a newly formed cell wall during cytokinesis. During this stage, organelles are released from the equatorial zone and are slowly dispersed in the cytoplasm to occupy the cellular spaces between the four distinct nuclei, hence forming a transient X-shaped organelle structure (Rodkiewicz 1988). This re-positioning of organelles at the end of meiosis II could potentially be coordinated by the radial microtubule arrays (RMAs) that are formed by the physical interconnection of microtubule arrays emanating from four haploid daughter nuclei. Immediately after organelle displacement at telophase II, a row of small vesicles is formed in place of the equatorial band, which then assemble to initiate formation of the cell wall, thereby physically separating the four cytoplasmic domains of resulting daughter cells (Figure 1). Through this intrinsic synchronization of spatial organelle dynamics and regulation of cell division, dispersed organelles at MII become physically separated leading to a balanced segregation into each of the four resulting spores.

## Membrane tethering of mitochondria and other organelles during meiosis

Correct and stable inheritance of most organelles during meiotic cell division, similar as in mitosis, is maintained by their active localization within the cell, so that they can be equally assimilated into each daughter cell. During mitosis in yeast and mammals, mitochondria remain tethered to the plasma membrane *via* a specific protein complex; the Mitochondria-ER-Cortex Anchor or the MECA (Table 1) complex (Ping, Kraft et al., 2016). MECA is a member of a specific type of protein complexes, also known as tethers, which form membrane-specific anchoring regions for different types of organelles. The MECA complex directly interacts with mitochondria and the plasma membrane (PM), and this is achieved through two distinct lipid-binding domains in one of its major protein components, namely Num1, which has been identified in *Saccharomyces cerevisiae* (Ping, Kraft et al., 2016). More specifically, a membrane binding site within the N-terminal region of Num1 physically connects to the outer membrane of the mitochondrion, whereas a pleckstrin homology domain within the Num1 C-terminus interacts with the PI_4,5_PI_2_ lipid messenger in the plasma membrane (Ping, Kraft et al., 2016). During mitosis of budding yeast Num1 maintains stable contacts between mitochondria and cell cortex, and the tethering function of Num1 is essential for a balanced distribution of mitochondria at the end of the mitotic cell division (Lackner 2019). Interestingly, studies in yeast have revealed that the physical tethering of mitochondria to the cell membrane in early stage meiosis is also mediated by the MECA complex, with evidence for a functional role of some specific components. In budding yeast the Mcp5 homologue has been identified as an important subunit of the MECA complex and this anchoring protein revealed a particular function in tethering the mitochondria to the 4 cell poles during meiosis. Another MECA component identified in *S. cerevisiae*, namely Mdm36, has a proposed involvement in the successful linking of mitochondria and the ER to the cell cortex, but its specific molecular function has not yet been revealed (Sawyer, Joshi et al., 2019). Currently, potential orthologs of MECA components have not been identified in plants. However, the presence of a similar tethering mechanism in multiple eukaryotic species implies that plants most likely utilize a similar pathway to mediate organelle targeting and dynamics at the plasma membrane in early meiotic stages.

By clustering the organelles physically together at specific regions in the cell periphery, tether complexes establish a physical connection between the different organelles in a local subcellular context and thus facilitate their organization at the cellular level. The importance of this organelle tethering in meiotic cell division was demonstrated in yeast and mammals, revealing not only distinct functions in intracellular signalling, but also in organelle inheritance (Nhek, Ngo et al., 2010; Prinz 2014; Sawyer, Joshi et al., 2019). In budding yeast meiosis, this tethering helps the mother cell to retain a set of mitochondria after cell division, and thus contributes to controlling organelle inheritance towards the daughter cells (Lackner, Ping et al., 2013). Additionally, it is hypothesized that nuclear tethering of mitochondria and plastids *via* the formation of direct intermembrane bridges with the nuclear envelope in prophase I facilitates selection of healthy organelles, hence establishing a ‘purification’ mechanism to guarantee inheritance of intact organelles to the daughter cells (Sheahan 2007). Defected mitochondria and plastids are not tethered to the NE and are subsequently degraded through autophagy. This idea is supported by the fact that the four daughter cells all together only inherit half of the mitochondria and plastids that were initially present in the meiotic mother cell (Brewer and Fangman 1980; Sheahan 2007). In plant meiosis targeted localization of organelles to the plasma membrane (PM) has also been found to occur during prophase I with dynamic movement along the cell periphery, indicating for the presence of one or more PM (Table 1) tethering mechanisms (see Figure 1). However, the underlying molecular mechanism for this targeted PM localization has not yet been revealed, nor is it clear whether this also occurs at the nuclear envelope and whether it stimulates organelle connectivity or has other specific functions in plant meiosis.

In plant meiocytes with a simultaneous type of cytokinesis, the tethered mitochondria at the poles become detached from the cell cortex towards the end of meiosis I, and form an organelle group together with plastids and other small organelles at the central midzone of the meiocytes (Nebenfuhr, Frohlick et al., 2000; Sawyer, Joshi et al., 2019). In yeast meiosis, a similar detachment of organelles is observed at the start of MII, and it is hereby suggested that this is controlled by specific genetic regulators that govern the meiotic cell cycle, or alternatively that this is regulated within the overall framework of the cell cycle program. For example, in the absence of *NDT80*, a meiosis-specific transcription factor in yeast, meiotic cells not only show a delayed pachytene stage and a complete failure to enter into the MI division program, but in parallel also exhibit a remaining localization of mitochondria at the cell cortex (Cheng, Otto et al., 2018; Sawyer, Joshi et al., 2019).

**FIGURE 2:**
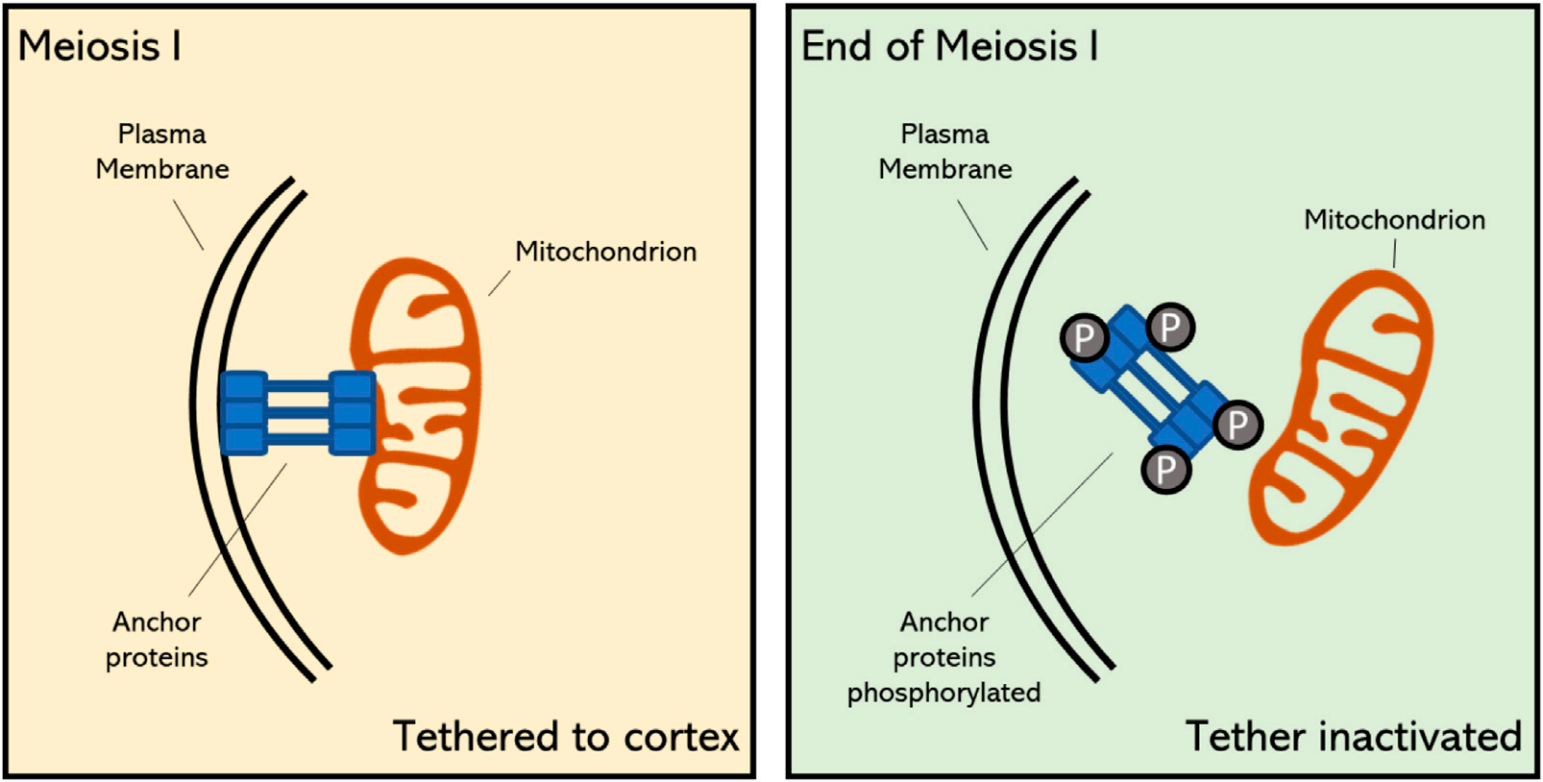
Proposed mechanism of mitochondrial tethering to the cell cortex during early stage meiosis and its detachment at the final stage of meiosis I in plants. The timely meiosis-specific detachment of mitochondria is caused by the destabilization of specific anchor proteins through phosphorylation (Sawyer, Joshi et al., 2019). Upon this programmed detachment, mitochondria become positioned in the cellular midzone and contribute to the formation of the organelle band.

The programmed release of mitochondria from the plasma membrane at the end of meiosis I is a specific feature of reductional cell division, as throughout mitotic cell division and the majority of meiosis I mitochondria remain associated with the cell cortex (Figure 2) (Chacko, Mehta et al., 2019; Sawyer, Joshi et al., 2019). This shows that meiotic cells have adopted a reprogrammed organization of mitochondrial dynamics and positioning throughout the cell cycle, and that the coordinated release in the cytoplasm at the end of MI is substantial for organelle inheritance or for other cellular functions in MII (Chacko, Mehta et al., 2019; Sawyer, Joshi et al., 2019). The programmed detachment of mitochondria from the PM is mediated *via* the timely inhibition of organelle-associated anchor proteins. In meiosis of budding yeast (*Saccharomyces cerevisiae*) it has been demonstrated that the MECA complex becomes destabilized at the earlier stages of meiosis II due to active phosphorylation of its subunits Num1 and Mdm36 by the meiosis-specific kinase Ime2 (Nishimura, Fukagawa et al., 2009). Whether PM tethering of mitochondria in early stages of plant meiosis is also mediated *via* a similar MECA complex-like anchoring mechanism and whether its programmed release at the end of MI is also mediated through phosphorylation of key proteins within the tethering complex is still not known.

In somatic plant cells, plastids have also been found to exhibit PM-directed localization, similar as mitochondria, however these organelles are sometimes found to be anchored in place *via* their stromules, i.e. small stroma-filled tubules that originate from the plastid surface and that occur in all plastid types (Hanson and Conklin 2020). Stromules have been observed to closely associate with other organelles, such as mitochondria and the ER, and are hypothesized to form a physical link to enable subcellular exchange of substrates and to carry out biological functions, as well as to accomplish specific signalling and anchoring purposes (Hanson and Conklin 2020). However, how this anchoring exactly works, and whether this also operates during meiotic cell division to target plastids to the PM or other subcellular regions is not yet known (Hanson and Sattarzadeh 2008).

## Cytoskeleton-dependent mobility of mitochondria and other organelles

During plant meiosis, several organelles show a distinct spatio-temporal organization at different stages, indicating for the presence of an active mechanism that governs their subcellular transfer and repositioning throughout the cell cycle. In general, organized movement of organelles is mainly regulated *via* cytoskeletal figures and associated actin and microtubule functions. As one of the best studied organelles, transport mechanisms of mitochondria are relatively well known, and this involves targeted mobility *via* several types of cytoskeletal structures (Heggeness, Simon et al., 1978; Boldogh and Pon 2006; Chacko, Mehta et al., 2019). In plants, however, insights into mechanisms of mitochondria movement is mostly based on comparisons with other cellular structures, like chromosomes, and eukaryotic organisms. Studies in *A. thaliana* revealed that, similar as for chromosome segregation, the dynamic behaviour of mitochondria during both mitotic and meiotic cell division is based on tightly regulated movements along the cytoskeleton (Van Gestel, Kohler et al., 2002; Sheahan, Rose et al., 2004). In line with this, there are accumulating reports that suggest that mitochondrial movement in somatic plant cells mainly occurs *via* F-actin filaments, and additionally relies on microtubule-based interactions to promote their positioning (Liebe 1994; Zhao 1999; Van Gestel, Kohler et al., 2002). In contrast, studies in *Nicotiana tabacum* protoplasts showed that mitochondrial redistribution and segregation during mitotic cell division is dependent upon actin, but not upon tubulin filaments, indicating that each cell type or stage in the cell cycle program has a specific mechanistic array for enabling subcellular dynamics and segregation of mitochondria (Sheahan, Rose et al., 2004). Overall, as can be inferred from various reports, the level of involvement of each type of filament, i.e. tubulin- or actin-based, in the regulation of mitochondrial positioning can potentially differ between species and even between different tissues or cell types (Ball and Singer 1982; Brown and Lemmon 2000; Boldogh and Pon 2006; Breviario, Giani et al., 2013).

**FIGURE 3:**
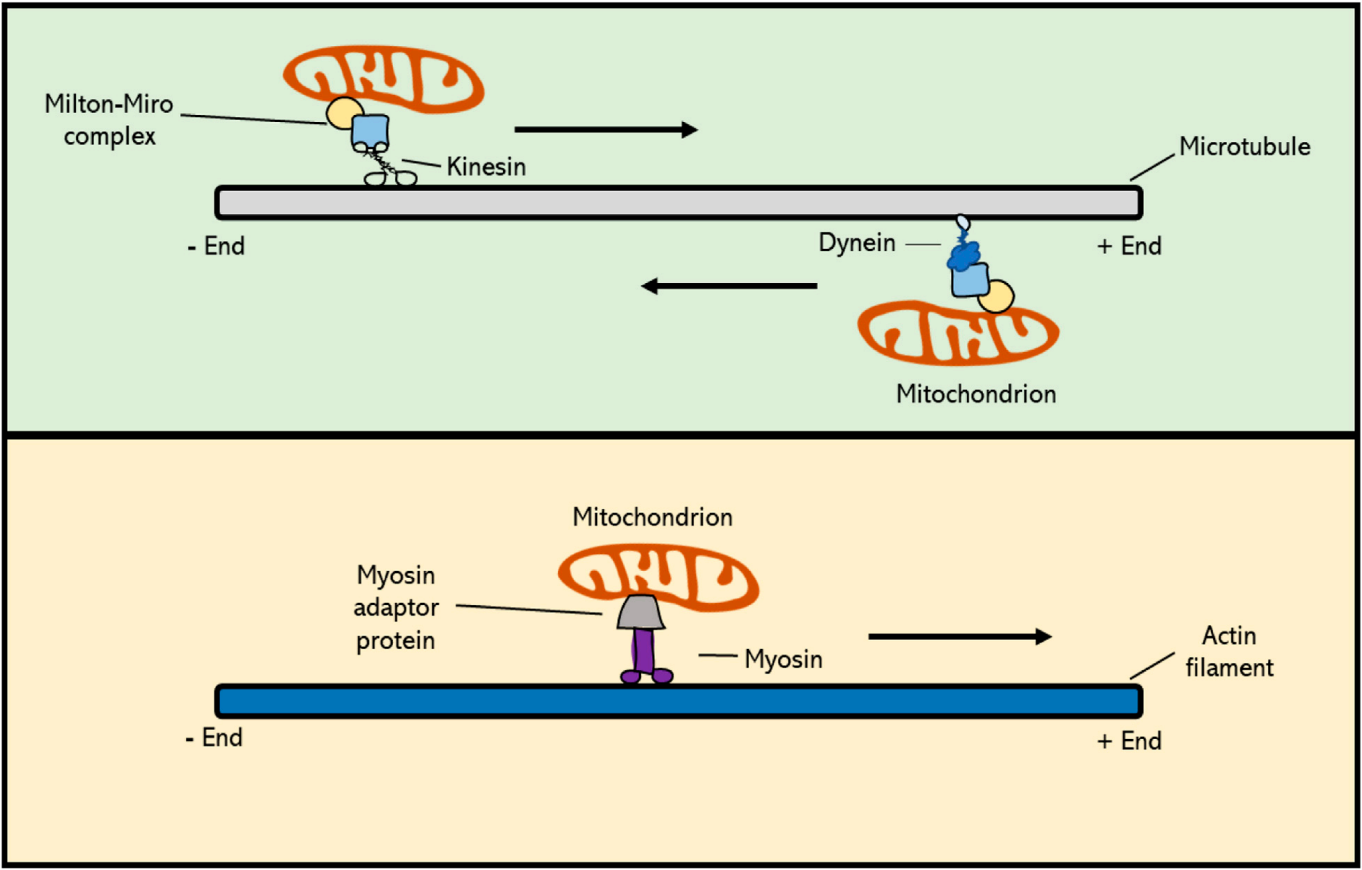
Cytoskeleton-based transport of mitochondria occurs *via* molecular motor proteins. Kinesin family proteins mediate cargo transport towards the plus-end of the MT polymer, whereas dyneins actively move toward the minus-end. In plant cells, MT-based movement of intracellular components is mediated by kinesins, as higher plants do not possess dyneins. Mitochondrial Rho GTPase proteins (Miro) form a complex with Milton adaptor proteins to facilitate the binding of mitochondria to motor proteins. Myosin motor proteins facilitate transport of cargo along the actin cytoskeleton, and this occurs *via* a potential, yet unknown, actin-specific adaptor protein. Myosins generally move towards the plus-end of F-actin, with the exception of myosin class VI, which moves towards the minus-end.

Actin filaments, also referred to as F-actin or actin fibres, are linear polymers of globular actin (G-actin) monomers and are present in the cytoskeleton as thin filaments. Actin filaments have a dual function in the cell. At the one side, they provide contractile forces to confer structural changes in the cell, whereas at the other hand they function as a cytoskeletal array to guide intracellular transport and movement of organelles and proteins (Kandasamy and Meagher 1999; Duan, Li et al., 2020; Strzyz 2021). In eukaryotes actin is thought to contribute to mitochondrial segregation, both in mitotic and meiotic cell division. However, to what extent actin is hereby actually involved is not yet clear. Overall, the subcellular movement of mitochondria in plant cells can be classified into two types: namely 1) directional movement, in which mitochondria travel long distances towards a specified region, and 2) wiggling, which is used for short distance transfer. It has recently been shown that long distance movement of mitochondria in Arabidopsis mesophyll cells depends on and is mediated by F-actin filaments (Oikawa, Imai et al., 2021). Also, in studies using Arabidopsis root hairs, it has been shown that the bidirectional movement and turnaround of mitochondria is dependent upon circular F-actin bundles (Zhang, Sheng et al., 2014). Moreover, genetic defects and application of chemical inhibitors that destabilize actin filaments result in defected orientation and movement of mitochondria in cultured tobacco cells (*Nicotiana tabacum* L.), similar as occurs for vesicles, the vacuole, Golgi and RNA (Boldogh and Pon 2006). In line with this, mitochondria have been found to co-localize with actin cables during both plant mitosis and meiosis, similar as was observed for mammals and yeast (Van Gestel, Kohler et al., 2002; Boldogh and Pon 2006; Strzyz 2021). In yeast, there is direct evidence for the role of actin filaments in mitochondrial inheritance during meiotic cell division, with time-lapse imaging revealing a specific antero- and retrograde movement of mitochondria along the actin cables (Fehrenbacher, Yang et al., 2004). In plants, however, little is yet known about the functional role of actin filaments in mitochondrial dynamics and transmission. In general, transport of cellular components along actin and microtubule (MT) filaments is mediated *via* motor proteins; i.e., molecular transporters that bind with specific cargo molecules and that use ATP hydrolysis to move along cytoskeletal filaments. Myosin family proteins, which are actin-based motor proteins, have been shown to play an important role in mitochondrial motility during mitosis in mammals and yeast (Boldogh and Pon 2006; Course 2016). However, of more than 30 classes of myosins found in eukaryotes, plants only encode two of them: myosin VIII and myosin XI, meaning that relatively to animals, plants contain fewer myosins and thus likely rely much less on actin-based organelle moment and cell organization (Nebenfuhr and Dixit 2018). Myosin VIII, a motor protein unique to plants, has a N-terminal neck region that consists of only three IQ motifs, which function as major calcium sensors, and a coiled-coil region that is shorter compared to that of myosin XI (Wang and Pesacreta 2004; Nebenfuhr and Dixit 2018). However, the functional implication of this structural difference is currently not known, as there are relatively few studies on myosin VIII in flowering plants. In somatic plant cells, myosins have been found to be involved in the intracellular organization and motility of mitochondria (Liebe 1994; Zhao 1999; Nebenfuhr and Dixit 2018). Studies in maize (*Zea mays* L.) revealed a co-localization of myosin XI with mitochondria and plastids, suggesting that myosin motors could mediate transport of organelles along the actin filament network in plants (Wang and Pesacreta 2004). However, whether the myosin-actin motility system acts as the main mechanism for subcellular organelle movement in plants, and if so, whether myosin directly attaches to the mitochondria or requires a myosin-specific adaptor is still unknown (Figure 3). Moreover, although generally presumed to operate in plant somatic cells, it is yet not determined whether the F-actin-myosin system plays a similar role in the dynamic organization of mitochondria in plant meiosis.

Microtubule filaments, which are formed by the polymerization of α- and β-tubulin subunits, as well as γ-tubulin at the minus ends, are also involved in regulating mitochondrial motility during mitotic cell division in plants by creating a cytoskeletal scaffold that mediates their targeted transport (Breviario, Giani et al., 2013). This was initially derived from cytological studies showing subcellular co-localization of mitochondria and microtubules, and was further evidenced by treatments with microtubule (MT) depolymerizing drugs that resulted in misalignment and imbalanced segregation of mitochondria during and after cell division, respectively (Heggeness, Simon et al., 1978; Ball and Singer 1982). Directed transport of mitochondria and other cellular components along MT filaments is mediated by two families of molecular motors; i.e. kinesins, that generally move towards the plus-end side of microtubules, and dyneins that promote transport towards the minus-end. Studies in mammalian placental cells revealed that the deletion of a kinesin-1 family member, KIF5B, results in a significant redistribution of the mitochondria, leading to a clustered accumulation in the cell (Ni, Wang et al., 2005; Chen and Sheng 2013). In *Arabidopsis thaliana* root cells, the kinesin-like protein AtKP1 was found to co-localize with mitochondria during many cellular processes, most likely indicating for the existence of a similar mechanism of mitochondrial positioning in plants (Ni, Wang et al., 2005). Moreover, *via* immunostaining assays, it was shown that AtKP1 co-localizes with several other organelles in mitotic interphase, though not with specific microtubule arrays formed in mitosis. This suggests that the AtKP1 kinesin is involved in spatial organization of mitochondria, but not through the general microtubule arrays, like the spindle or the phragmoplast (Ni, Wang et al., 2005). Contrary to AtKP1, two other Arabidopsis kinesins, namely the minus-end-directed kinesin 14 family proteins ATK1 and ATK5, have been found to localize to the meiotic and mitotic spindles. In the absence of ATK1, both mitotic and meiotic spindles exhibit multipolarity, as caused by an insufficient translocation of microtubules to the spindle poles, eventually resulting in abnormal chromosome segregation (Nebenfuhr and Dixit 2018). Other cell division defects originating from the functional loss of kinesin family proteins include misoriented cell plates, leading to irregular cell division patterns, and defects in cell plate expansion, leading to incomplete cytokinesis in both somatic cells and gametophytes (Strompen, El Kasmi et al., 2002; Tanaka, Ishikawa et al., 2004; Oh, Bourdon et al., 2008). However, despite clear alterations in chromosome segregation and cell division, putative effects of the absence of ATK1 or other kinesin types on organelle positioning have not been described in mitosis nor in meiosis. Dyneins are minus-end directed microtubule motor proteins and form an important family of MT motor proteins in animals (McGrath 2005). However, higher plants lack dyneins and the segregation of intracellular components during plant mitotic and meiotic cell division therefore relies entirely on kinesins (Lambert 1993). All together, these studies suggest that kinesin family proteins, and thus plus-end directed mobility along microtubules, are crucial for the correct dynamic behaviour and spatial distribution of mitochondria in the cell (Mishra and Chan 2014). However, it is yet unclear which type of MT fibres is used for this active organelle transport in plant meiosis, and *via* which molecular system this is exactly mediated. Mitochondrial Rho (Miro) proteins are members of the small GTPase protein family that are highly conserved across most eukaryotes, including plants, and that play a role in mitochondrial motility. They contain a transmembrane domain that physically connects with mitochondria by integrating into the outer mitochondrial membrane. Moreover, Miro proteins, as seen in *C. elegans*, form a complex with Milton adaptor proteins, which directly link to kinesin and dynein molecular motors (Figure 3) (Shen, Ng et al., 2016). This binding between the Miro-Milton complex and MT-based motor proteins is Ca^2+^-dependent. When calcium levels in the surrounding environment are high, the Miro-Milton complex releases the attached kinesin, which is then bound to syntaphilin (SNPH) molecules. These SNPHs block the ATPase activity of kinesin and hence inhibit the movement of mitochondria (Chen and Sheng 2013). When Ca^2+^ levels are low, the Miro-Milton complex is tightly linked to the kinesin and therefore remains its ATPase activity to promote intracellular motility of mitochondria. In the *A. thaliana* genome three putative Miro orthologs have been identified (Yamaoka, Nakajima et al., 2011)*.* Two of these, namely *MIR O 1* and *MIR O 2*, are expressed ubiquitously in all types of plant tissues, with their encoded products specifically co-localizing with mitochondria. Genetic studies revealed that AtMIRO1 is essential for embryonic cell division, i.e. with corresponding mutants showing a significantly reduced level of mitochondria in the two-cell embryo stage, suggesting that Miro proteins play a major functional role in the intracellular distribution of mitochondria in plant cells (Yamaoka, Nakajima et al., 2011). Both meiosis and mature pollen grains appear normal in the Arabidopsis *miro1* mutant, indicating that MIRO1 is not essential for meiotic cell division and microspore development. Despite the preliminary insights into the relevance and molecular control of MT-based mitochondrial dynamics in mitotic cell division in plants, further research is needed to fully characterize the extent and functional role of MT-based motility of mitochondria and other organelles in plant meiosis, as well as its regulation by MIROs and other factors.

There is accumulating evidence that most organelles in the plant cell, and especially membrane-bound organelles, similar to mitochondria, are actively transported within the cytoplasm *via* a cytoskeleton-based mechanism (Brownfield, Yi et al., 2015; Duan, Li et al., 2020; Mathur 2020). In tobacco (*Nicotiana tabacum* L.) it has been demonstrated that both the random and directed movement of non-green plastids in epidermal leaf cells is entirely halted when the cells are treated with chemical inhibitors of microfilament and -tubule biosynthesis (Kwok and Hanson 2003). Also, in *Arabidopsis* leaf cells, actin inhibitor treatments not only cause loss of actin localization around chloroplasts but also lead to a severe disorganization of the chloroplast arrays (Kandasamy and Meagher 1999). These reports suggest that both actin and tubulin have critical importance for the mobility and subcellular organization of various types of plastids in somatic plant cells, and thus may mediate the movement of these organelles in meiotic cell division as well. Moreover, largely based on the structural similarity between mitochondria and plastids, it is presumed that active transport of plastids along cytoskeletal figures is also carried out by specific motor proteins, such as myosins and kinesins (Breviario, Giani et al., 2013). However, further research is needed to verify whether intracellular transport of plastids and other organelles in the plant cell actually occurs *via* the cytoskeleton, and if so, to unravel the specific molecular mechanisms that mediate this targeted transport. Moreover, although it is generally presumed that the programmed organelle dynamics in plant meiosis relies on similar cytoskeletal mechanisms, a detailed study of the actin and microtubule figures throughout meiosis and their contribution in the targeted localization of mitochondria and other organelles throughout the meiotic cell cycle needs to be performed.

## A functional role for organelles and organelle structures in plant meiosis

The spatial organization and dynamics of all types of cytoplasmic organelles during meiotic cell division in plants is essential to guarantee balanced inheritance to the resulting daughter cells. In the context of meiosis, organelles therefore have long been viewed as passive components that merely require to be equally distributed across the four haploid spores, but that do not play any role in meiotic regulation. Recent findings, however, have led to a paradigm shift by providing accumulating evidence that organelles and organelle-based structures have adopted specific cellular functions in plant meiotic cell division and contribute to the regulation of key processes, such as determination of cell polarity, configuration of microtubule organizing centres (MTOCs), and orientation of MII spindles. Additionally, various studies have reported that mitochondria associate with energy consuming structures like chloroplasts and the endoplasmic reticulum during meiosis, suggesting that mitochondria could be an important source of energy provision for meiotic cell cycle progression and related processes (Logan 2010).

## Plastids establish meiotic MTOCs in basal plant systems to determine cell polarity and spindle positioning in MI and MII

Plant cells, unlike animals, lack centrioles and therefore use alternative structural mechanisms to assign cell polarity and to indicate the initiation sites of spindle biogenesis, thereby intrinsically establishing spatial directionality of chromosome segregation and cell division. These structures define the subcellular regions at which microtubule filaments are synthesized and from which they emanate to generate the spindle, and therefore are referred to as MicroTubule Organizing Centers or MTOCs. Interestingly, in plant meiosis determination and spatial assignment of the four MTOCs is largely mediated by plastids, with two major mechanisms described depending on the evolutionary level of the organism. Basal plant systems, including charophytes, bryophytes (only in a few hepatics), and lycopsid pteridophytes (e.g. Marattiales), exhibit a monoplastidic type of meiotic cell division (Brown and Lemmon 1990; Renzaglia 1993; Brown 2001). This type of meiosis is seen as a plesiomorphic feature that was inherited from algal ancestors containing a single plastid and that further developed during plant evolution. In this system, as described for liverworts, the nascent sporocyte or archesporal meiotic progenitor cell contains only one single plastid that undergoes two successive divisions at the onset of meiosis to form four plastids that are positioned in a tetrahedral arrangement in the cytoplasm (Renzaglia 1993). These four plastids define the sporocyte’s cell polarity, as reflected by its subsequent development into a quadrilobed shape, and specifically serve as MTOCs for structurally organizing subsequent spindle formation and cell division (Kosetsu, Murata et al., 2017). More specifically, upon double plastid division in mid-prophase I (or sometimes later in some taxa), the four polar plastids serve as distinct sites for MT nucleation and thus generate four cones of microtubules radiating from the poles to form a unique quadripolar microtubule system (QMS) (Brown and Lemmon 1997). This complex plastid-MT configuration is highly dynamic throughout meiotic cell division and transforms into a typical bipolar-shaped spindle array in metaphase I to mediate reductional chromosome segregation. As seen in *Angiopteris*, coinciding with the breakdown of the nuclear envelope, spindle-like structural arrays originate from the four plastid tips, and interact with kinetochores to produce a tetrapolar-like MI spindle. Following this, the plastids are surrounded by SER and are clustered per two, so that eventually a bipolar metaphase I spindle is physically established between two pairs of polar plastids (Renzaglia 1993; Brown 2001). After MI, the plastid-MT complex regains its tetrahedral configuration and microtubular cones emanating from opposite plastids interact with each other to form the two perpendicularly oriented spindles in metaphase II that guide equational chromosome segregation (Brown and Lemmon 1997). Importantly, parallel to guiding chromosomes in MI and MII, the QMS (Table 1) array determined by the plastids also functions as a structural scaffold along which the motility of several other cellular components takes place (Shimamura, Brown et al., 2004). As such, the quadripolarity established by the four plastids in early meiosis in basal land plants guarantees that each spore receives a plastid and a haploid nucleus, and also contributes to the inheritance of other organelles.

Higher land plants, including more evolved ferns as well as all seed plants, have adopted a poly-plastidic type of meiosis. In this type of meiotic cell division program, the sporocyte mother cells harbour multiple plastids that exhibit a highly organized spatio-temporal localization throughout meiosis I and II to guarantee balanced organelle segregation (Kosetsu, Murata et al., 2017). The polyplastidic type of meiosis also relies on the formation of MTOCs as initiation sites for the biogenesis of spindles in MI and MII, however, these specific structures and the associated cell polarity are not established by plastids. In general, MTOCs in polyplastidic meiocytes are more diffuse, and their exact identity and structural composition is still not yet resolved. Cytological studies in a range of plants with a polyplastidic type of meiosis, such as the liverwort *Ricciocarpus natans* (Brown and Lemmon, 2000), indicate that MTOCs are generated by local nucleation of γ-tubulin, and that the nuclear surface takes up this microtubule (MT) nucleating function in both meiosis and mitosis (Lambert 1993; Kosetsu, Murata et al., 2017). It was thereby reported that NE-bound nucleation of γ-tubulin in meiotic prophase I initially leads to multiple MTOCs that construct a multipolar meiotic spindle-like structure which then further transforms into a distinct bipolar spindle in Metaphase I *via* directed clustering of MTOCs at opposite poles of the meiocyte. However, how this polar positioning of γ-tubulin-based MTOCs in MI is mediated, and how the tetrahedral cell polarity in MII is established is currently not yet known. Overall, since plastids or any other type of organelle do not localize at the MTOCs during meiosis in higher plants, it is presumed that the basal function of plastids in determining meiotic cell polarity is lost and is taken up by another mechanism, most likely relying on local nucleation of γ-tubulin.

## Mid-cellular organelle band safeguards MII spindle positioning in simultaneous-type of meiosis

Male meiosis in dicotyledonous plants typically exhibits a simultaneous-type of cytokinesis, with cell wall formation only occurring at the end of meiosis II in between the four generated haploid daughter nuclei. Because of this, the two haploid chromosome sets generated in meiosis I are not physically separated in two distinct daughter cells, but instead remain located in the same cytoplasm, though with a distinct localization at both poles. In order to guarantee a maximum physical separation of the four nuclei at the end of meiosis II, the two spindles in metaphase II are consistently organized in a perpendicular manner to hence obtain a tetrahedron-oriented separation of haploid nuclei (Busby 1988). Initially it was assumed that this tightly regulated perpendicular positioning of MII spindles was determined by a programmed imposition of spindle pole MTOCs in early-stage meiosis. However, genetic studies in the model system *Arabidopsis thaliana* have revealed that this spatial regulation of MII spindles is actually determined by meiotic organelles, and in particular by the organelle band that is positioned between the two segregated chromosome sets in MI and that remains located at the central midzone throughout meiosis II. More specifically, loss-of-function mutants of the *Arabidopsis thaliana* protein JASON (JAS) were found to exhibit an altered, more randomized positioning of MII spindles in male meiosis, thereby resulting in the ectopic formation of tripolar, parallel and even fused spindles and associated events of meiotic restitution (d'Erfurth, Jolivet et al., 2008; De Storme and Geelen 2011). Strikingly, meiotic chromosome preparations additionally revealed that male meiocytes of the *jason* mutant lack the internuclear organelle band during meiosis II, suggesting that the positioning of the organelles at the central midzone establishes a mechanistic framework to impose the perpendicular orientation of spindles in Metaphase II. In addition, it was found that JASON effectively associates with vesicles in the organelle band and also co-localizes with the endomembrane, indicating that JASON is effectively needed to confer vesicle transfer or maintain central organelle positioning during meiosis II, and that this is essential for correct positioning of the spindles in Metaphase II (Brownfield, Yi et al., 2015). Parallel to defects in MII organelle band formation, the *jason* mutant also exhibits alterations in organelle clustering in meiosis I. Instead of grouping on opposite sides of the cell, the organelles were found to be dispersed within the cytoplasm. Overall, these data demonstrate that JASON contributes to organelle positioning in *Arabidopsis* male meiosis and that alterations herein have an impact on the positioning of the MII spindles, leading to defects in chromosome segregation and reductional division. However, interestingly, localization experiments in *jason* male meiocytes revealed an equal distribution of organelles after telophase II, even when the organelle band is completely lacking, indicating that the formation of the organelle band in MII is not essential for balanced organelle inheritance in plant meiosis (Brownfield, Yi et al., 2015). The mechanism by which JASON controls organelle positioning in male meiosis I and II, and how this affects the biogenesis or orientation of MII spindles and associated chromosome segregation still requires further investigation.

## The nucleus-cytoplasm interaction contributes to meiotic cell cycle regulation in plants

In plants, like in other eukaryotic organisms, the cytoplasmic state continuously evolves together with the corresponding nuclear genome in order to maintain a balanced compatible interaction between the organellar (mtDNA and cpDNA) and the nuclear DNA to hence safeguard the cell’s metabolism and related processes. Specific alterations in this equilibrium state are referred to as alloplasmy, i.e., typically defined as the combination of a nuclear genome with another ‘foreign’ cytoplasm, and this often occurs in plants resulting from a broad range of natural and artificial processes, such as interspecific and -generic hybridization, allopolyploidy, somatic hybridization, and haploid induction. Alloplasmy in plants often leads to ‘genetic incompatibilities’ or a skewed interaction between the cytoplasmic and the nuclear genome, and therefore may lead to alterations in plant development and physiology with often strong phenotypic deviations from the original parent lines. In many cases this also results in severe reproductive defects or sterility and this is either due to homeotic alterations in floral organogenesis, as in *Triticum aestivum* L. x *Aegilops crassa* L. alloplasmic lines (Murai 1993; Kianian and Kianian 2014; Ogihara, Takumi et al., 2015), or is caused by a decreased level of ATP/ADP and reduced energy supply in flower buds, as in the alloplasmic *Nicotiana tabacum* L. *x Nicotiana repanda* L. F1 hybrids (Bergman, Edqvist et al., 2000). Moreover, studies in durum wheat (*Triticum turgidum* L.) have shown that alloplasmy significantly affects the integrity of the parental mitochondrial genomes, resulting in distinct heteroplasmy caused by structural rearrangements and nucleotide changes. Overall, this suggests that the alloplasmic status promotes mutagenesis and recombination in the mtDNA and hence forms one of the main factors driving mitochondrial genome evolution (Noyszewski et al., 2014). Importantly, alloplasmy has also been found to interfere with the overall progression of the meiotic cell cycle or the integrity of specific meiotic processes in plants, indicating that a compatible nucleus-cytoplasm interaction is needed for a regular meiotic cell division program. For example, studies using *Brassica rapa* L. x *Diplotaxis muralis* L. alloplasmic lines revealed that male meiosis in resulting F1 lines is substantially delayed, hence forming a mechanistic basis for the associated male sterility (Yamasaki 2014). In parallel, alloplasmic lines of maize (*Zea mays spp. mays*) that contain teosinte cytoplasm exhibit structural alterations in meiocyte organization with the occurrence of two physically separated chromosome clusters in MI that show a slightly asynchronous progression of chromosome dynamics together with the occasional presence of extra nucleoli (Poggio, Rosato et al., 1997). Moreover, studies using alloplasmic F1 lines resulting from the hybridization of common wheat (*Triticum aestivum L.*) with rye (*Secale cereale* L.) or with hexaploid triticale revealed that the alien cytoplasm influences the meiotic chromosome behaviour in MI with a particular impact on the extent of homo (eo)logous pairing in prophase I and induction of meiotic restitution (Wang 1999). Overall, these findings demonstrate that the cytoplasmic status of the cell, i.e. as determined by the genomic configuration of the organelle pool, has a major impact on the genomic stability and integrity of the enclosed organelles and additionally controls patterns of meiotic recombination and meiotic cell cycle progression with alloplasmy often conferring distinct irregularities herein.

## Conclusion

Prior to the development of technologies such as confocal/electron microscopy and organelle probes, it was widely believed that organelle inheritance was a simple and purely arbitrary event, as there was little evidence to support otherwise. With new technological advances, it has now been revealed that organelle inheritance is actually a highly controlled and structurally regulated process, that is governed by mechanisms that ensure the transition of healthy and sufficient organelles to the progeny cells. Research into the specifics of organelle dynamics in plant meiosis is still preliminary, and current insights are mainly based on similar mechanisms as recorded in different organisms. However, it is well documented that plant cells, including meiocytes, differ from other eukaryotic cell types by the evolutionary adoption of some important elements, such as plant-specific organelles (i.e. chloroplasts), distinct sub-cellular structures, and the mechanism of energy production. It is therefore highly expected that some of these attributes, together with the cytoskeletal arrays as determined by actin and/or microtubule functions, contribute to the maintenance and mobility of organelles in plant meiosis. Overall, further cytological analyses and genetic approaches are required to characterize the spatial organization of the different types of organelles throughout meiosis I and II, and to reveal the specific mechanisms that underlie this programmed behaviour as well as its functional role in organelle inheritance and putatively other processes in plant meiosis.
